# Supplementary figures and images for: Clusterization in head and neck squamous carcinomas based on lncRNA expression: molecular and clinical correlates
Source: Clin Epigenetics. 2017 Apr 8;9:36. doi: 10.1186/s13148-017-0334-6 (PMC5385094; doi:10.1186/s13148-017-0334-6)

Figure S1

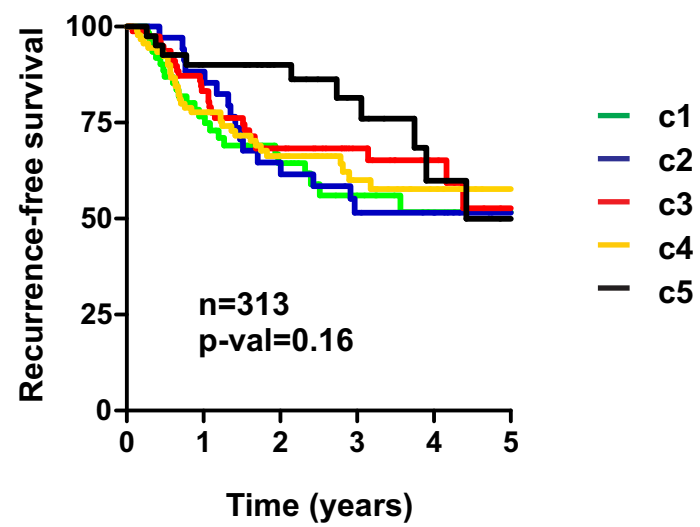

Supplement: Supplementary file 2 — Kaplan-Meier plot of TCGA HNSCC patients stratified by lncRNA clusters using recurrence as endpoint. P val was calculated with the log-rank test. n: number of patients with available follow-up information. (PDF 472 kb) [file 13148_2017_334_MOESM2_ESM.pdf]
